# Supplementary material for: Genetic Basis of Pigment Dispersion Syndrome and Pigmentary Glaucoma: An Update and Functional Insights
Source: Genes (Basel). 2024 Jan 23;15(2):142. doi: 10.3390/genes15020142 (PMC10887877; doi:10.3390/genes15020142)
Supplement: Supplementary file 1 [file genes-15-00142-s001.zip › genes-2778182-supplementary.pdf]

Supplementary materials

Table S1. Seach strategies used in identifying relevant publications

Table S2. Genome-wide association studies and candidate gene studies of pigment dispersion syndrome and pigmentary glaucoma

Table S1. Seach strategies used in identifying relevant publications

**Search order**	**Query**	**Results**
1	(Medical Genetics OR genotype OR genetics[Subheading] AND genetics)	4,116,691
2	"pigmentary glaucoma"[All Fields] OR "glaucoma related pigment dispersion syndrome"[Supplementary Concept] OR "glaucoma related pigment dispersion syndrome"[All Fields] OR "pigment dispersion syndrome"[All Fields] OR ("pigmentary"[All Fields] AND "glaucoma"[All Fields]) OR "pigmentary glaucoma"[All Fields])	858
3	1 AND 2	83

Table S2. Genome-wide association studies and candidate gene studies of pigment dispersion syndrome and pigmentary glaucoma

	First Author	Year	Phenotype	Sample Size	Genetic analysis	Study Population	Genes	Significant association	Ref
**Genome-wide association study**									
1	Simcoe, M. J.	2022	PDS/PG	Cases (574), controls (52,627)	GWAS	European	GSAP, GRM5/TYR	Yes	[1]
**Candidate gene study**									
1	Fingert, J. H.	2016	PG	PG (209), no controls	qPCR assay, CNV	Caucasian	*TBK1*	No	[2]
2	Giardina, E.	2014	PDS/PG	Patients (84), controls (200)	GA	Caucasian	*LOXL1*	No	[3]
3	Wolf, C.	2010	PG	PG (88), controls (280)	GA	Caucasian	*LOXL1*	No	[4]
4	Rao, K. N.	2008	PDS/PG	PG (44), PDS (34), controls (108)	GA	Caucasian	*LOXL1*	No	[5]
5	Lynch, S.	2002	PG	PG probands from 4 families	Sequencing of *TYRP1* gene	Caucasian	*TYRP1*	No	[6]
6	Jaksic, V.	2010	PG	1 Patient	Case report	Caucasian	MTHFR C677T	na	[7]

CNV, copy number variants; GA, genetic case-control association study; GWAS, Genome-Wide Association Meta-Analysis; na, not applicable; PDS, Pigment Dispersion Syndrome; PG, Pigmentary Glaucoma; qPCR, Quantitative Polymerase Chain Reaction

References

1. Simcoe, M.J.; Shah, A.; Fan, B.; Choquet, H.; Weisschuh, N.; Waseem, N.H.; Jiang, C.; Melles, R.B.; Ritch, R.; Mahroo, O.A.; et al. Genome-Wide Association Study Identifies Two Common Loci Associated with Pigment Dispersion Syndrome/Pigmentary Glaucoma and Implicates Myopia in its Development. *Ophthalmology* **2022**, *129*, 626-636, doi:10.1016/j.ophtha.2022.01.005.
2. Fingert, J.H.; Robin, A.L.; Scheetz, T.E.; Kwon, Y.H.; Liebmann, J.M.; Ritch, R.; Alward, W.L. Tank-Binding Kinase 1 (TBK1) Gene and Open-Angle Glaucomas (An American Ophthalmological Society Thesis). *Transactions of the American Ophthalmological Society* **2016**, *114*, T6.
3. Giardina, E.; Oddone, F.; Lepre, T.; Centofanti, M.; Peconi, C.; Tanga, L.; Quaranta, L.; Frezzotti, P.; Novelli, G.; Manni, G. Common sequence variants in the LOXL1 gene in pigment dispersion syndrome and pigmentary glaucoma. *BMC ophthalmology* **2014**, *14*, 52, doi:10.1186/1471-2415-14-52.
4. Wolf, C.; Gramer, E.; Müller-Myhsok, B.; Pasutto, F.; Gramer, G.; Wissinger, B.; Weisschuh, N. Lysyl oxidase-like 1 gene polymorphisms in German patients with normal tension glaucoma, pigmentary glaucoma and exfoliation glaucoma. *Journal of glaucoma* **2010**, *19*, 136-141, doi:10.1097/IJG.0b013e31819f9330.
5. Rao, K.N.; Ritch, R.; Dorairaj, S.K.; Kaur, I.; Liebmann, J.M.; Thomas, R.; Chakrabarti, S. Exfoliation syndrome and exfoliation glaucoma-associated LOXL1 variations are not involved in pigment dispersion syndrome and pigmentary glaucoma. *Molecular vision* **2008**, *14*, 1254-1262.
6. Lynch, S.; Yanagi, G.; DelBono, E.; Wiggs, J.L. DNA sequence variants in the tyrosinase-related protein 1 (TYRP1) gene are not associated with human pigmentary glaucoma. *Molecular vision* **2002**, *8*, 127-129.
7. Jaksic, V.; Markovic, V.; Milenkovic, S.; Stefanovic, I.; Jakovic, N.; Knezevic, M. MTHFR C677T homozygous mutation in a patient with pigmentary glaucoma and central retinal vein occlusion. *Ophthalmic research* **2010**, *43*, 193-196, doi:10.1159/000272023.
